# Synergistic Regulation of Pigment Cell Precursors’ Differentiation and Migration by *ednrb1a* and *ednrb2* in Nile Tilapia

**DOI:** 10.3390/cells14151213

**Published:** 2025-08-06

**Authors:** Zilong Wen, Jinzhi Wu, Jiawen Yao, Fugui Fang, Siyu Ju, Chenxu Wang, Xingyong Liu, Deshou Wang

**Affiliations:** 1 Integrative Science Center of Germplasm Creation in Western China (Chongqing) Science City, Southwest University, Chongqing 400715, China; wzl787500@email.swu.edu.cn (Z.W.); z980171878@email.swu.edu.cn (J.W.); g093615@email.swu.edu.cn (J.Y.); ffg2022@email.swu.edu.cn (F.F.); 17362494986@163.com (S.J.); wolfgang0625@163.com (C.W.); 2 Key Laboratory of Freshwater Fish Reproduction and Development (Ministry of Education), Key Laboratory of Aquatic Science of Chongqing, School of Life Sciences, Southwest University, Chongqing 400715, China

**Keywords:** *ednrb*, pigment cell precursors, synergistic regulation, guanine, erythrophores, heritable

## Abstract

**Highlights:**

1. *ednrb1a−/−*; *ednrb2−/−* mutants exhibit complete iridophore loss, similarly to mpv17 mutants.
2. *ednrb* mutants display no defects in the guanine synthesis pathway in tilapia.
3. *ednrb1a* and *ednrb2* synergistically regulate iridophore and erythrophore development.
4. *mitfa* mRNA injection rescues the phenotype of *ednrb1a−/−*;*ednrb2−/−* mutants.

**Abstract:**

The evolutionary loss of *ednrb2* in specific vertebrate lineages, such as mammals and cypriniform fish, raises fundamental questions about its functional necessity and potential redundancy or synergy with paralogous endothelin receptors in pigment cell development. In teleosts possessing both *ednrb1a* and *ednrb2* (e.g., Nile tilapia), their respective and combined roles in regulating neural crest-derived pigment cell precursors remains unresolved. Using CRISPR/Cas9, we generated single and double *ednrb* mutants to dissect their functions. We demonstrated that *ednrb1a* and *ednrb2* synergistically govern the differentiation and migration of iridophore precursors. While *ednrb1a* is broadly essential for iridophore development, *ednrb2* plays a unique and indispensable role in the colonization of iridophores in the dorsal iris. Double mutants exhibit near-complete iridophore loss; severe depletion of melanophores, xanthophores, and erythrophores; and a striking, fertile, transparent phenotype. Crucially, this iridophore deficiency does not impair systemic guanine synthesis pathways. mRNA rescue experiments confirmed *mitfa* as a key downstream effector within the Ednrb signaling cascade. This work resolves the synergistic regulation of pigment cell fates by Ednrb receptors and establishes a mechanism for generating transparent ermplasm.

## 1. Introduction

Endothelin receptors (Ednrs) belong to the rhodopsin-like receptor family of G-protein-coupled receptors and mediate physiological functions by binding to endothelin ligands. Although an Ednr-like gene has been identified in the cephalochordate amphioxus, it lacks conservation with vertebrate Ednrs, and their ligand-encoding genes are exclusive to vertebrates. Thus, the endothelin system is considered vertebrate-specific [1]. Cyclostomes (e.g., lampreys) retain a single *ednrb*, while cartilaginous fishes (e.g., elephant sharks) and certain tetrapods possess two paralogs, *ednrb1* and *ednrb2*. Teleosts, having undergone three rounds of whole-genome duplication, typically retain three paralogs: *ednrb1a*, *ednrb1b*, and *ednrb2*. However, *Ednrb2*/*ednrb2* has been lost in mammals (excluding Prototheria) and zebrafish, though the evolutionary mechanisms underlying this loss remain unclear [2,3]. It remains to be empirically validated whether the loss of *ednrb2* is restricted to zebrafish or extends to other Cyprinidae species, or even across the entire Cypriniformes order. Furthermore, in specific Cypriniformes and Salmoniformes lineages that have undergone four rounds of genome duplication, it remains unclear whether *ednrb2* has been completely lost or whether novel paralogs have arisen via subfunctionalization or neofunctionalization. Systematic investigation of these evolutionary dynamics is required to resolve the genomic fate and functional diversification of *ednrb2* in teleosts.

Previous studies demonstrate that Ednra primarily regulates craniofacial skeletal development. Mice with Ednra mutations exhibit severe craniofacial malformations and cardiovascular outflow tract defects, with homozygotes dying at birth due to respiratory failure secondary to craniofacial abnormalities [4]. In contrast, Ednrb is critical for pigment cell development. In tetrapods, studies demonstrate that Ednrb is critically involved in melanocyte development. In birds, neural crest cells (NCCs) residing in the migration staging area (MSA) prior to their lateral migration pathway exhibit reduced Ednrb1 expression but elevated Ednrb2 expression, with Ednrb2 maintaining consistent expression throughout melanocyte differentiation [5]. However, no functional reports of Ednrb2 exist in osteichthyans (bony fish). In zebrafish, Ednrb1a is expressed in melanophores, iridophores, xanthophores, and their precursor cells [6]. The absence or loss of Ednrb2 in some mammals and zebrafish raises questions about its evolutionary significance. If Ednrb2 is functionally redundant with Ednrb1a in pigment cell development, as seen in tetrapods, its absence in osteichthyans might compromise pigment pattern formation. Alternatively, this loss could indicate that Ednrb1a alone suffices for pigment cell development in these lineages, suggesting functional divergence or compensatory mechanisms unique to ray-finned fish (Actinopterygii).

Research on Ednrb in fish species remains limited. Through studies on the zebrafish *rose* mutant, the mutation site has been identified in the *ednrb1a* gene on chromosome 1, with exon analysis revealing that a G-to-A transition in exon 6 introduces a premature termination codon, halting translation early. While the mutant exhibits normal pigment patterns in early development, melanophores and iridophores begin decreasing by 21 dpfs (days post fertilization), and adult mutants retain only approximately half the melanophores and iridophores of wild-type individuals, indicating normal initial development of these cells but severe developmental impairment later. Further studies reveal that *ednrb1a* is expressed during late embryogenesis, metamorphic pigment pattern formation, and throughout embryonic development. *ednrb1a* is expressed in melanophores, xanthophores, iridophores, and their precursor cells. Unlike endothermic vertebrates that have a single neural crest-derived pigment cell type, melanocyte, ectothermic vertebrates possess six classes of pigment cells, melanophores, xanthophores, erythrophores, iridophores, leucophores, and cyanophores, collectively referred to as chromatophores [7,8,9,10,11]. Teleosts exhibit color patterns generated by the arrangement of specific pigment cells [12,13]. Model fish species such as medaka (*Oryzias latipes*) and zebrafish (*Danio rerio*) possess three types of chromatophores: melanophores, xanthophores, and iridophores [14]. Melanophores, which are responsible for black pigmentation, contain melanin and represent the ectotherm counterpart of mammalian melanocytes. Xanthophores display yellow or orange coloration due to carotenoids and pteridines. Iridophores produce iridescence through purine-rich reflecting platelets [15]. Unlike medaka and zebrafish, cichlids possess a fourth pigment cell type, erythrophores, alongside melanophores, xanthophores, and iridophores. This expanded repertoire contributes to their diverse body colors and patterns. Nile tilapia (*Oreochromis niloticus*), a cichlid species of significant importance in global aquaculture [16,17], has emerged as a prominent model system for investigating pigment pattern formation and chromatophore biology [18,19].

In this study, we established *ednrb* mutants of Nile tilapia using CRISPR/Cas9 technology. Through experimental approaches including guanine content quantification in skin, fluorescence in situ hybridization (FISH), quantitative real-time PCR (qPCR), phylogenetic analysis, synteny analysis, amino acid sequence alignment, and mRNA rescue, we revealed that *ednrb* mutations disrupt iridophore precursor cell differentiation, leading to iridophore depletion and concomitant reductions in melanophores, erythrophores, and xanthophores, ultimately resulting in a transparent body. These findings provide novel insights into Ednrb functionality in teleosts and establish a critical model for genetic breeding innovation in aquaculture species.

## 2. Materials and Methods

### 2.1. Animals

The Nile tilapia (*Oreochromis niloticus*) used in this study were obtained from the Key Laboratory of Freshwater Fish Resources and Development (Ministry of Education) at Southwest University (Chongqing, China). All fish were maintained at 26 °C in recirculating aquaculture systems under natural photoperiod conditions. Offspring were generated by artificial fertilization, and fertilized eggs were incubated at 26 °C in a recirculating water incubator to obtain fry. All animal experiments were conducted in strict compliance with the *Guide for the Care and Use of Laboratory Animals* and were approved by the Experimental Animal Ethics Committee of Southwest University (No. IACUC-20230308).

### 2.2. Phylogenetic and Amino Acid Sequence Alignment Analyses

Amino acid sequences of Ednrb from representative vertebrate taxa human (*Homo sapiens*), mouse (*Mus musculus*), chicken (*Gallus gallus*), lizard (*Anolis carolinensis*), spotted gar (*Lepisosteus oculatus*), Japanese (*Oryzias latipes*), Nile tilapia, and three-spined stickleback (*Gasterosteus aculeatus*) were retrieved from the NCBI database (https://www.ncbi.nlm.nih.gov/) (Table S1). Sequence alignment was performed using MEGA-X software 11.0.13 and saved in MEGA format, and a Neighbor-Joining phylogenetic tree was constructed with 1000 bootstrap replicates to assess nodal reliability, followed by visualization refinement in Adobe Illustrator 2022. For sequence conservation analysis, ClustalW alignment of selected species was conducted via MegAlign software 17.3.1, with pairwise sequence similarity quantified using distances in amino acid sequences of Ednrb from representative vertebrate taxa retrieved from the NCBI database. Sequence alignment was performed using MEGA-X software and saved in MEGA format, and a Neighbor-Joining phylogenetic tree was constructed with 1000 bootstrap replicates to assess nodal reliability, followed by visualization refinement in Adobe Illustrator 2022. For sequence conservation analysis, ClustalW alignment of selected species was conducted via MegAlign software, with pairwise sequence similarity quantified using sequence distances. Conserved residues and domains were identified using GeneDoc 2.7 and the NCBI Conserved Domain Search tool v.1.54, respectively, and conserved domains were annotated on the alignment using Adobe Illustrator 2022. This integrated approach elucidates evolutionary relationships and the structural–functional conservation of Ednrb across vertebrates.

### 2.3. Quantitative Real-Time PCR (qPCR)

Total RNA was extracted from adult tilapia tissues (*n* = 9) and samples of fish at 90 dpfs (*n* = 20) using RNAiso reagent (Takara, Kasukabe, Japan). RNA concentration was measured using a Nanodrop 2000 spectrophotometer (Thermo Fisher Scientific, Wilmington, DE, USA). Reverse transcription and cDNA synthesis were performed using the PrimeScript^®^ RT reagent kit with gDNA Eraser (Takara, Japan). The synthesized cDNA was diluted 10-fold and used as the template for quantitative real-time PCR (qPCR). qPCR was conducted following the manufacturer’s instructions for the TB Green^®^ Premix Ex Taq™ II kit. Relative gene expression levels were calculated using the 2^−∆∆Ct^ method, with *gapdh* serving as the internal reference gene. Specific primer sequences for the target genes are listed in Table S2.

### 2.4. Mutation of ednrb1a,ednrb2 and Establishment of Homozygotes

Mutation targets were designed in exon 5 (encodes transmembrane domain, and comprehensive screening of upstream exons failed to yield suitable target sequences) of *ednrb1a* and exon 2 of *ednrb2* in tilapia, with the target sequences CCATTTTCTACACCCTGATGACC for *ednrb1a* and CCACCCTCGCAGCCAGGCCGACC for *ednrb2*, using the online website (http://crispr.dbcls.jp/). CCA is the PAM region. Cas9-mRNA (1000 ng/μL) and guide-RNA (500 ng/μL) were mixed evenly at a ratio of 1:1, and phenol red was added as an indicator at a total volume of 1/10. Then the mixture was injected into zygotes by microinjection to obtain F0 chimera of tilapia *ednrb1a*, *ednrb2* mutants. F0-generation chimeras were intercrossed to generate F1 progeny that are either single heterozygous (mono-het) or double heterozygous (double-het). Priority was given to crossing double-het F1 males and females to produce F2-generation double homozygous mutants. Concurrently, single-het F1 individuals were retained as backups for line establishment. The primers used for gDNA synthesis were as follows:

*ednrb1a*-gRNA-F: TAATACGACTCACTATAGGTCATCAGGGTGTAGAAAAGTTTTAGAGCTAGAAATAGC
*ednrb2*-gRNA-F: TAATACGACTCACTATAGGTCGGCCTGGCTGCGAGGGGTTTTAGAGCTAGAAATAGC
*ednrb1a* -gRNA-R: AGCACCGACTCGGTGCCAC
*ednrb2* -gRNA-R: AGCACCGACTCGGTGCCAC

### 2.5. Phenotype Analysis and Chromatophore Counting

Wild-type (WT) fish, *ednrb1a*^−/−^*,ednrb2*^−/−^ and *ednrb1a*^−/−^*;ednrb2*^−/−^ mutants (*n* = 10/per genotype), were photographed at 5, 7, 17, 30, 90, and 180 dpfs. Fish within 30 dpfs were photographed using a Leica M165FC stereomicroscope (Leica, Wetzlar, Germany), while adult fish were photographed using a Nikon D7000 camera (Nikon, Tokyo, Japan). The morphology and number of melanophores in the skin vertical bars, inter bar, caudal fins, and scales of mutants at 90 dpfs were analyzed by taking photos and counting. To enhance melanophore visibility during counting, fish were anesthetized in 4.5 mg/mL tricaine methanesulfonate [20,21] (MS-222; Western Chemical, Inc., Ferndale, WA, USA), followed by immersion in 10 mg/mL L-epinephrine hydrochloride (Sigma-Aldrich, St. Louis, MI, USA) for 15 min to induce chromatophore contraction. Then, surgical scissors were used to cut off 25 mm^2^ sized fins and placed them on a glass slide and photographed using an Olympus microscope BX53 (Olympus, Shinjuku, Japan).

### 2.6. H.E. Staining

Larvae of *ednrb1a*^−/−^*, ednrb2*^−/−^ and *ednrb1a*^−/−^*;ednrb2*^−/−^ mutants and WTs at 14 days were fixed overnight in Bouin’s fluid at 4 °C, followed by washing in 70% ethanol (replaced 2–3 times over 24 h) to remove excess picric acid. For hematoxylin and eosin (H.E.) staining, fixed samples were dehydrated through a graded ethanol series (70%, 80%, 95%, and 100%, 1 h each), cleared in xylene (twice, 30 min each), and embedded in paraffin wax (6 0 °C, three changes, 1 h each). Serial 5 μm sections were cut using a microtome, mounted on slides, and dried overnight at 37 °C. Sections were deparaffinized in xylene (twice, 10 min each), rehydrated through a descending ethanol series (100% to 50%, 5 min each), and stained with hematoxylin for 5–8 min. After rinsing in tap water, differentiation was performed in 1% acid ethanol (5–10 s), followed by bluing in 0.2% ammonia water (1–2 min). Counterstaining was carried out with 0.5% eosin for 1–2 min, followed by rinsing in distilled water [22,23]. Slides were dehydrated through an ascending ethanol series (70% to 100%, 2 min each), cleared in xylene (twice, 5 min each), and mounted with resinous medium under coverslips for microscopic analysis.

### 2.7. Fluorescence In Situ Hybridization

WT embryos at 3 days post-fertilization (dpfs) were dissected to remove the yolk sac, fixed overnight in 4% paraformaldehyde, embedded, and sectioned. On the first day, sections were dewaxed in xylene, rehydrated through a graded ethanol series, and rinsed three times in 1× PBS, and endogenous peroxidase activity was blocked with 3% H_2_O_2_ (30 min, room temperature (RT)) [24,25]. Samples were treated with proteinase K (5 min, 37 °C), post-fixed in 4% PFA (5 min, 4 °C), and washed in 1× PBS, and the background was reduced using triethanolamine. After two washes in 1× PBST (PBS + 0.1% Tween-20), sections were pre-hybridized in buffer at 65 °C for 3 h [26]. A hydrophobic barrier pen was used to outline the tissue area, and DIG-labeled RNA probes diluted in hybridization buffer were applied to fully cover the sections, followed by overnight hybridization at 65 °C. On the second day, sections were washed in graded SSCT buffers (2× to 0.2× SSC), cooled to RT, rinsed in 1× PBS and DIG wash buffer (DIGI), and blocked for 3 h at RT. Anti-DIG horseradish peroxidase (HRP) antibody (1:2000 dilution) was applied and incubated overnight at 4 °C. On the third day, sections were washed three times in DIGI and 1× PBS, and signal amplification was performed using tyramide signal amplification. After PBS rinses, nuclei were counterstained with DAPI (15 min, RT), mounted with antifade medium, and imaged immediately using the Olympus FV3000 Confocal Laser Scanning Microscope (Olympus, Tokyo, Japan).

### 2.8. Elisa (Enzyme-Linked Immunosorbent Assay)

Skin tissues were selected from WTs and *ednrb* mutants at 30 dpfs and weighed to 0.2 g fresh weight using a precision balance. The tissues were placed in 1.8 mL of phosphate-buffered saline PBS with a pH of approximately 7.2, homogenized under cold conditions using a low-temperature homogenizer, and centrifuged at 4 °C at 5000 rpm for 15 min to separate the supernatant [27]. The guanine content in the supernatant was measured using a guanine enzyme-linked immunosorbent assay ELISA kit (Meimian, Yancheng, China) following the manufacturer’s instructions.

### 2.9. mitfa and pnp4a mRNA Rescue

The *mitfa* and *pnp4a* ORF (open reading frame) was PCR-amplified from Nile tilapia skin cDNA using primers designed with the following modifications: the forward primer incorporated a T7 promoter sequence (5′-TAATACGACTCACTATAGGG-3′) at its 5′ end, while the reverse primer included the SV40 3′UTR sequence containing the polyadenylation signal. The PCR product was A-tailed and ligated into the pMD-19T simple vector. Following the transformation of DH5α competent cells, positive clones were selected and validated by Sanger sequencing. Correct recombinant plasmids were designated pMD-19T-T7-*mitfa*-SV40 and pMD-19T-T7-*pnp4a*-SV40 [28,29]. The insert was re-amplified by PCR from this plasmid and used as template for in vitro transcription using the mMESSAGE mMACHINE T7 ULTRA Kit (Thermo Fisher Scientific, Waltham, MA, USA) to generate capped mRNA. Approximately 500 ng of mRNA was microinjected into the yolk of single-cell stage zygotes derived from *ednrb1a*^−/−^*;ednrb2*^−/−^ mutants.

### 2.10. Statistic Analysis

Values are presented as the mean ± SD. One-way ANOVA, followed by Duncan’s multiple comparison, was used to determine the significance of differences among different groups. All data were analyzed using GraphPad Prism 8 (version 8.01, GraphPad Software, San Diego, CA, USA). *p* < 0.05 was considered to be statistically significant.

## 3. Results

### 3.1. Comparative Analysis of Ednrb Amino Acid Sequences in Vertebrates

The alignment of Ednrb amino acid sequences across vertebrates revealed conserved structural features, including a signal peptide, seven-transmembrane (7 TM) domains characteristic of G-protein-coupled receptors, and endothelin ligand binding sites (Figure S1) [30,31,32]. Transmembrane helices 1–3 and 7, along with their extracellular loops, mediate interactions with the C-terminal region of endothelin ligands, facilitating ligand–receptor binding and signal transduction. In contrast, helices 4–6 influence ligand selectivity by interacting with distinct C-terminal ligand motifs. The C-terminal domain of Ednrb undergoes palmitoylation and phosphorylation, modifications that likely modulate receptor coupling to distinct G-protein subtypes. Following the third round of whole-genome duplication (3R), functional divergence among Ednrb paralogs via subfunctionalization or neofunctionalization may have altered ligand–binding specificity or intracellular signaling pathways [33,34,35].

Sequence similarity analyses (Table S1) demonstrated that Nile tilapia Ednrb1a shares 61.5–76.1%, 15.0–36.4%, and 85.4–97.6% identity with tetrapod Ednrb1 of in full-length sequence, signal peptide, and ligand binding sites, respectively. Strikingly, tilapia Ednrb1a exhibits higher similarity to the other teleost Ednrb1a (90.4–91%, 68.2%, and 97.6% for the same regions). In contrast, tilapia Ednrb1b shows lower identity to tetrapod Ednrb1 (51.9–58.3%, 6.2–31.2%, and 75.6–87.8%), suggesting that teleost Ednrb1a retains stronger functional conservation with ancestral Ednrb1 than Ednrb1b. Similarly, tilapia Ednrb2 shares 61.8–72.6%, 11.8–37.5%, and 92.7–97.6% identity tetrapod Ednrb2, while displaying elevated similarity to other teleost Ednrb2 (80.8–83.8%, 58.8–82.4%, and 97.6%). These results indicate that teleost Ednrb1a and Ednrb2 have evolutionarily conserved roles analogous to their 2R vertebrate counterparts, whereas Ednrb1b may have undergone greater functional divergence post-3R [36,37,38,39].

### 3.2. Phylogenetic Analysis of Ednrb in Vertebrates

The Ednrb family has diversified in vertebrates through multiple rounds of whole-genome duplication [40]. The phylogenetic reconstruction of Ednrb across representative species reveals that most vertebrates retain two paralogs, Ednrb1 and Ednrb2 (Figure S2). Teleosts, having undergone a third round of WGD (3R), possess additional duplicates (Ednrb1a and Ednrb1b), whereas eutherian (e.g., human, mouse) and metatherian mammals (e.g., kangaroo) retain only *Ednrb1*, suggesting the secondary loss of *Ednrb2* in these lineages. In contrast, prototherian mammals (platypus *Ornithorhynchus anatinus*, echidna *Tachyglossus aculeatus*), non-mammalian tetrapods (chicken, lizard), and basal actinopterygians (paddlefish *Polyodon spathula*) retain both *Ednrb1* and *Ednrb2*, indicating ancestral retention of both paralogs in lineages predating 3R.

Expanding on prior reports of *ednrb2* loss in zebrafish, this study identifies the convergent loss of *ednrb2* in Cypriniformes species, including Chinese sucker (*Myxocyprinus asiaticus*), sharpnose sturgeon (*Acipenser oxyrinchus*), grass carp (*Ctenopharyngodon idella*), and goldfish (*Carassius auratus*). Conversely, *ednrb2* is retained in Characiformes (*Hyphessobrycon nattereri*, *Colossoma macropomum*), Siluriformes (*Pelteobagrus fulvidraco*), Anguilliformes (electric eel, *Electrophorus electricus*), Salmoniformes (rainbow trout, *Oncorhynchus mykiss*), and other teleosts (Nile tilapia, Japanese medaka, pufferfish *Takifugu rubripes*). These findings suggest *ednrb2* loss is widespread in Cyprinidae, Cobitidae, and Catostomidae (Cypriniformes). Notably, some Cypriniformes and Salmoniformes lineages with a fourth WGD (4R) exhibit the further duplication of *ednrb1a* into *ednrb1aa* and *ednrb1ab* (Figure S2).

### 3.3. The Expression Patterns of ednrb1a and ednrb2

**Figure 1:**
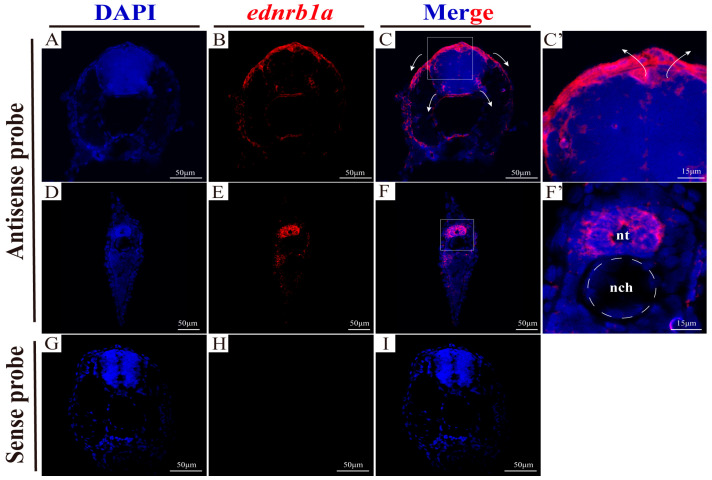
Expression localization of *ednrb1a* in trunk NCCs by FISH at 3 dpfs. (**A**–**C**) Expression localization of *ednrb1a* in trunk NCCs near the cephalic side. (**D**–**F**) Expression localization of *ednrb1a* in trunk NCCs near the caudal side. (**C′**,**F′**) Higher magnification of (**C**,**F**), respectively. (**G**–**I**) Negative controls with *ednrb1a* sense probes. The white arrows indicate migration paths of NCCs. nt, neural tube. nch, notochord.

Due to the early timing of NCC differentiation and migration, WT embryos at 3 and 4 dpfs were selected to investigate *ednrb* expression patterns via whole-mount in situ hybridization (WISH). The results revealed that *ednrb1a* was strongly expressed in the dorsal head region at 3 dpfs and subsequently spread along NCC migratory pathways toward the trunk by 4 dpfs (Figure S3D–J). *ednrb2* exhibited a similar spatial expression profile (Figure S3F–L), while *ednrb1b* showed negligible expression at both stages (Figure S3E–K). To further validate *ednrb* expression in NCCs, transverse sections of 3 dpfs WT embryos were analyzed using fluorescent in situ hybridization (FISH). *Ednrb1a* expression was prominent in trunk NCCs near the cranial region, which had detached from the neural tube and migrated along dorsolateral and ventral pathways to differentiate into derivatives (Figure 1A–C′) [41,42,43]. In caudal trunk regions, *ednrb1a* expression was restricted to NCCs within the neural tube, representing pre-epithelial–mesenchymal transition (pre-EMT) cell clusters (Figure 1D–F′) [44].

### 3.4. Establishment of Tilapia ednrb1a,ednrb2 and ednrb1a;ednrb2 Homozygous Mutants

Mutation targets were designed in exon 5 of *ednrb1a* and exon 2 of *ednrb2* in tilapia. CRISPR/Cas9 gene editing was used to mutate these genes (Figure S4A,B). After the F0 generation chimeras were sexually maturated, they were mated with wild-type XX female fish to obtain the F1 heterozygotes. Various types of F1 heterozygotes were identified by Sanger sequencing (Figure S4C,D). Through screening the F1 generation, we obtained many male and female tilapia with *ednrb1a* mutations (+7 bp and +13 bp insertions) and *ednrb2* mutations (−26 bp deletion), including *ednrb1a*^+7/+13^; *ednrb2*^−26/−26^ double mutants and *ednrb1a*^+2/+2^ single mutants (Figure S4C–E). Ultimately, F2-generation homozygous mutants were established, comprising *ednrb1a*^+7/+13^*,ednrb2*^−26/−26^, *ednrb1a*^+7/+13^; *ednrb2*^−26/−26^, and *ednrb1a*^+2/+2^ (Figure S4F–I). These mutations cause frameshift mutation and the premature termination of Ednrb translation (Figure S4C,D) [45].

### 3.5. Early-Stage Ednrb Mutants Exhibit Variable Iridophore Reduction in Tilapia

**Figure 2:**
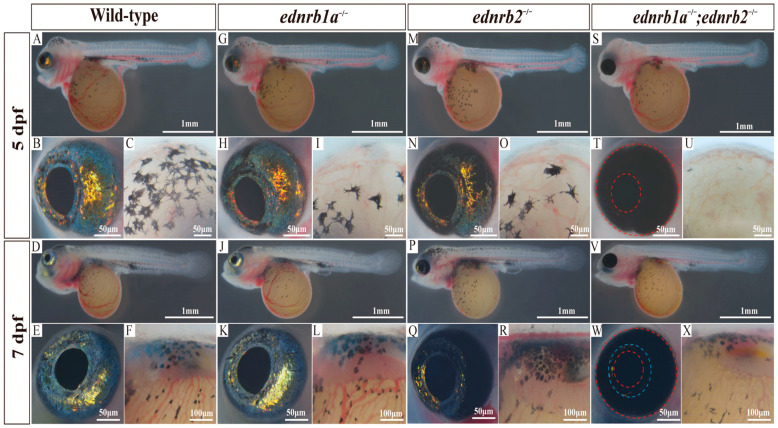
Phenotypes of WTs and homozygous mutants at 5 dpfs and 7 dpfs. (**A**–**F**) Phenotypes of WT (and magnified views of the eye, the head, and the ventral body, before and after). (**G**–**L**) Phenotypes of *ednrb1a*^−/−^ mutants. (**M**–**R**) Phenotypes of *ednrb2*^−/−^ mutant. (**S**–**X**) Phenotypes of *ednrb1a*^−/−^*;ednrb2*^−/−^ mutant. The red dashed lines represent the edges of the iris and RPE, and the blue dashed lines represent the remaining iridophores on the iris. dpfs, days post fertilization.

In WTs, dendritic melanophores were abundantly observed on the head by 5 dpfs, while iridophores were predominantly localized to the iris at this stage. By 7 dpfs, the ventral region of WT was progressively covered with iridophores (Figure 2A–F), enabling clearer assessment of iridophore loss in mutants. Larvae from WTs and mutants at 5 dpfs and 7 dpfs were analyzed. In *ednrb1a*^−/−^ mutants, the iris appeared normal, but melanophores on the head were reduced, and only sparse iridophores covered the ventral region (Figure 2G–L). *ednrb2*^−/−^ mutants exhibited dorsal iris iridophore loss, mild melanophore reduction in the head, and limited ventral iridophores (Figure 2M–R) [46,47]. Double mutants (*ednrb1a*^−/−^; *ednrb2*^−/−^) showed near complete iris iridophore loss [near the retinal pigment epithelium (RPE)] (Figure S5A–L), severe melanophore absence in the head, and transparent ventral regions due to iridophore deficiency (Figure 2S–X) [48,49]. By 17 dpfs, WTs developed distinct vertical black bars, abundant xanthophores in the head, and preliminary pigmentation patterns by 30 dpfs. Comparative analysis at these stages revealed that melanophores, iridophores, and xanthophores increased significantly in WTs, with iridophores imparting a bluish hue via light reflection; dorsal melanophores formed complexes with iridophores, extending dendritic processes around them. *ednrb1a*^−/−^ mutants displayed silvery-yellow skin with drastically reduced iridophores, and dendritic melanophores failed to form complexes, while *ednrb2*^−/−^ mutants showed transient iridophore reduction at 17 dpfs but recovered this to near the level of WT phenotypes by 30 dpfs, except for persistent iridophore loss of the dorsal iris. Double mutants were transparent with internal organs visible due to complete iridophore loss in the body and iris (Figure S6). Quantification confirmed significant xanthophore reduction in *ednrb1a*^−/−^ mutants and severe melanophore/xanthophore loss in double mutants.

### 3.6. Impact of Ednrb Mutations on Pigment Cells in Tilapia at 90 dpfs

**Figure 3:**
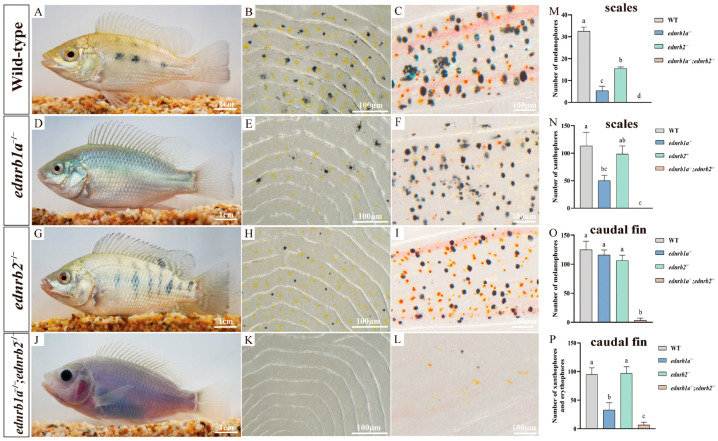
Phenotypes of WTs and homozygous mutants at 90 dpfs. (**A**–**L**) Phenotypes of WT, *ednrb1a*^−/−^*,ednrb2*^−/−^ and *ednrb1a*^−/−^*;ednrb2*^−/−^ mutants and higher magnification of dorsal scales and caudal fins. (**M**–**P**) Statistical analysis of melanophores, xanthophores and erythrophores in the dorsal scales and caudal fins of WTs, *ednrb1a*^−/−^*,ednrb2*^−/−^ and *ednrb1a*^−/−^*;ednrb2*^−/−^ mutants. Data are expressed as the mean ± SD (*n* = 5). Significant differences in the data were tested by one-way ANOVA followed by Tukey’s test. Different letters above the error bar indicate statistical differences. *p* < 0.05 is considered to be statistically significant. dpfs, days post fertilization**.** In panels (**A**–**L**), black and red boxes indicate the magnified regions of dorsal scales and caudal fins, respectively.

At 90 dpfs, the scales of WTs were densely populated with dendritic melanophores and ovoid xanthophores. The caudal fin exhibited melanophores displaying coarse-grained, punctate patterns, accompanied by irregularly shaped erythrophores ranging in color from orange to red (Figure 3A–C) [50]. In contrast, *ednrb1a*^−/−^ mutants displayed a silvery-green body coloration. Specifically, this manifested as: a significant reduction in the density of melanophores and xanthophores on the scales; melanophores in the caudal fin showed markedly smaller pigment granules, and erythrophores were drastically reduced in number, appearing only sporadically (Figure 3D–F). *ednrb2*^−/−^ mutants exhibited an overall pigmentation phenotype similar to WTs. However, the size of melanophores on the scales was significantly reduced (Figure 3G–I). Strikingly, *ednrb1a*^−/−^; *ednrb2*^−/−^ double mutants presented a transparent appearance. This resulted from a substantial reduction in melanophores, xanthophores, erythrophores, and iridophores across the body, including the scales and body wall [51,52]. Notably, in the iris region, the complete absence of iridophores led to the exposure of underlying dark structures, resulting in a black appearance (Figure 3J–L).

### 3.7. Ednrb Mutants Exhibit Ornamental Coloration in Tilapia at 180 dpfs

**Figure 4:**
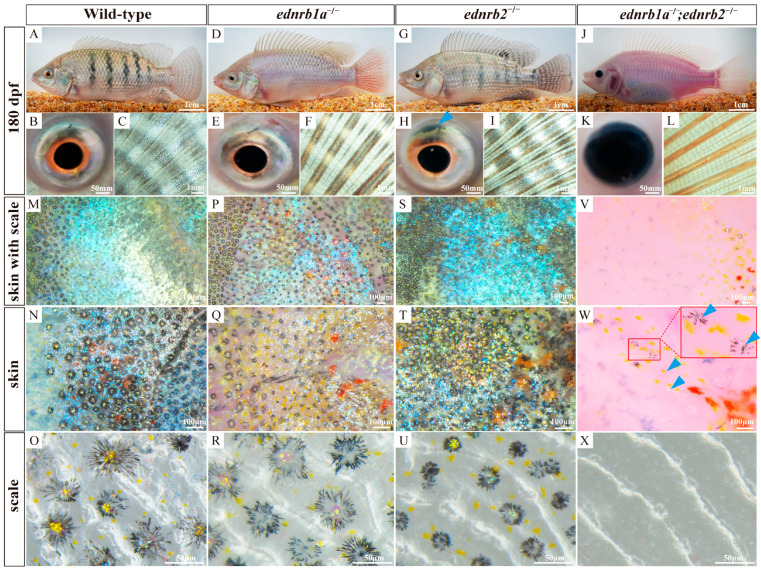
Phenotypes of WTs and homozygous mutants at 180 dpfs. (**A**–**L**) Phenotypes of WT, *ednrb1a*^−/−^*,ednrb2*^−/−^ and *ednrb1a*^−/−^*;ednrb2*^−/−^ mutants. Blue arrows indicate the loss of iridophores in iris. (**M**–**X**) Magnified views of the skin with scale, the skin without scale and the separated scale of WT, *ednrb1a*^−/−^*,ednrb2*^−/−^ and *ednrb1a*^−/−^*;ednrb2*^−/−^ mutants. The large red box was a magnified view of the small red box, and the blue arrows indicate the residual iridophores. dpfs, days post fertilization.

The WTs had very stable vertical bars and inter-bars at 180 dpfs (Figure 4A). The RPE and the iris were black (Figure 4B). The fins and scales were pigmented with many melanophores. Many xanthophores distributed in the gaps between the melanophores, and many iridophores gathered in spots (Figure 4C) [53,54]. In contrast, *ednrb1a*^−/−^ mutants exhibited a complete absence of vertical black stripes in both the skin and caudal fin. *Ednrb2*^−/−^ mutants displayed significantly faded skin stripes alongside intensified pigmentation in caudal fin stripes, accompanied by irreversible developmental defects in the dorsal iris. Double mutants (*ednrb1a*^−/−^;*ednrb2*^−/−^) presented a pinkish-transparent appearance across the body surface, with complete loss of black stripes and a dark pigmented iris (Figure 4D–L).

The scales of wild-type (WT) Nile tilapia were densely populated with melanophores overlaid by iridophores. These iridophores primarily aggregated into clustered structures at the central regions of melanophore clusters, reflecting blue light [55,56,57]. In contrast, the iridophore numbers on the scales of *ednrb1a*^−/−^ mutants were significantly reduced compared to those on WTs, causing underlying xanthophores and erythrophores to become exposed and visible. *ednrb2*^−/−^ mutants maintained iridophore distribution patterns and density equivalent to wild-type fish. Double mutants manifested near-complete loss of iridophores in the iris region alongside substantially reduced melanophores and xanthophores on the scales (Figure 4M–X).

### 3.8. Ednrb Mutants Display No Impairment of Guanine Synthesis

**Figure 5:**
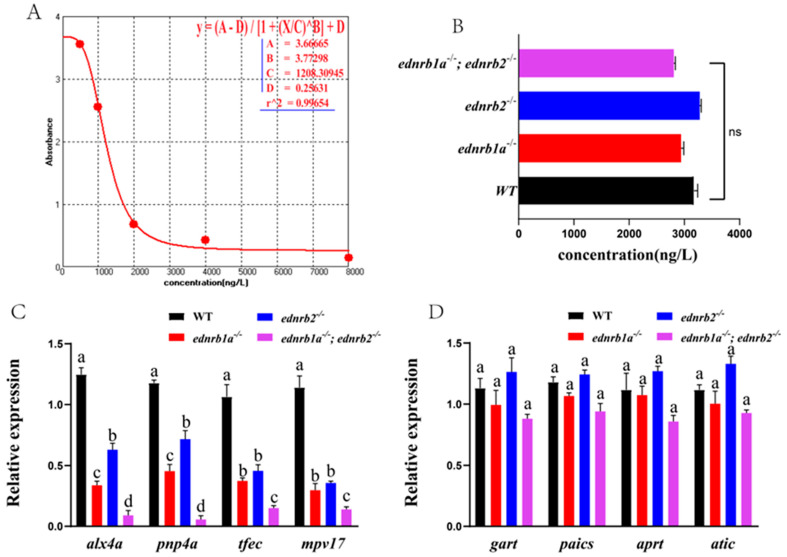
Integrated analysis of cutaneous guanine content in WTs and *ednrb* mutants at 30 dpfs. (**A**,**B**) Competitive ELISA standard curve for guanine quantification and cutaneous guanine content. (**C**,**D**) Expression levels of iridophore differentiation and pigmentation genes. Different letters above the error bar indicate statistical differences. *p* < 0.05 is considered to be sta-tistically significant. dpfs, days post fertilization.

The guanine content in the skin tissues of WTs, *ednrb1a*^−/−^*,ednrb2*^−/−^, and double mutants was quantified by ELISA (Note: Calculated using a standard curve where absorbance inversely correlated with guanine concentration) (Figure 5A). At 30 dpfs, no significant differences in guanine levels were observed between any mutant groups and WT (Figure 5B). Real-time qPCR analysis revealed that iridophore differentiation/migration-related genes (*alx4a*, *pnp4a*, *tfec*, *mpv17*) showed significant downregulation in mutants, with the most pronounced reduction in double mutants followed by *ednrb1a*^−/−^ (Figure 5C); guanine biosynthesis genes (*gart*, *paics*, *aprt*, *atic*) exhibited no significant expression changes between mutants and WTs (Figure 5D) [58,59,60,61,62]. While the loss of *ednrb1a* and *ednrb2* caused substantial iridophore depletion and disrupted iridophore differentiation, guanine biosynthesis and nucleotide metabolism remained unaffected [63,64,65,66].

### 3.9. Co-Expression of ednrb1a and ednrb2 in NCCs and Eyes

**Figure 6:**
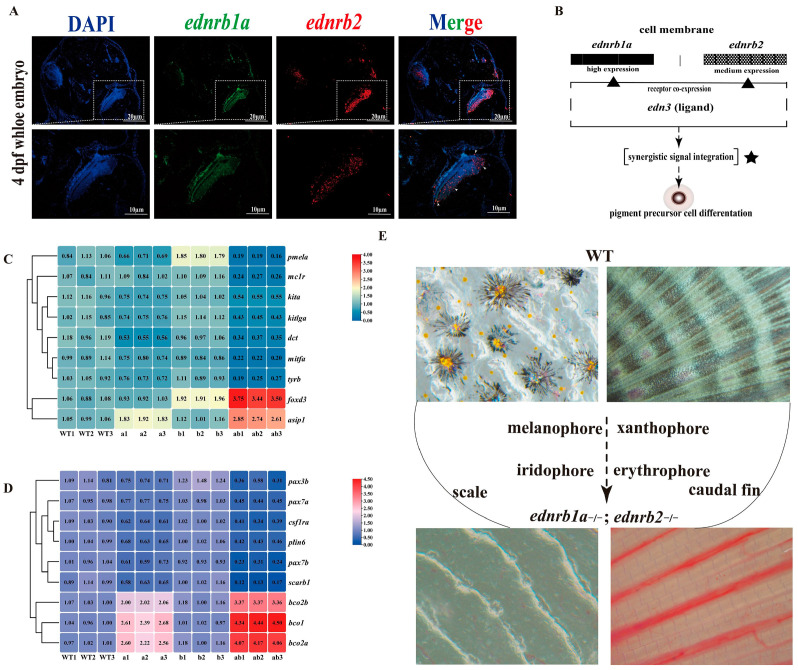
Co-expression of *ednrb1a* and *ednrb2* in trunk NCCs of WTs at 4 dpfs by FISH and *ednrb* mutation on pigment lineage differentiation/pigmentation. (**A**) Co-expression of *ednrb1a* and *ednrb2* in trunk NCCs and high-magnification views of the indicated regions. Blue: DAPI (nuclear counterstain); green: *ednrb1a*; red: *ednrb2*. (**B**) Schematic model of synergistic regulation of pigment precursor cell differentiation by *ednrb1a* and *ednrb2*. (**C**,**D**) Expression levels of genes related to melanophore and xanthophore differentiation, migration, and pigmentation in *ednrb* mutants. (**E**) Phenotypic manifestations in scales and caudal fins of WTs and *ednrb1a*^−/−^;*ednrb2*^−/−^ mutants at 150 dpfs.

Dual-color fluorescence in in situ hybridization revealed the co-expression of *ednrb1a* and *ednrb2* in NCCs and eyes in WTs at 4 dpfs (Figure 6A).

Real-time PCR analysis of skin samples from WTs, *ednrb1a*^−/−^*,ednrb2*^−/−^, and *ednrb1a*^−/−^;*ednrb2*^−/−^ mutants at 90 dpfs revealed significant dysregulation of pigment cell lineage differentiation and pigmentation-related genes. In melanophores, genes related to differentiation/migration (*mitfa*, *kita*, *kitlga*, *mc1r*) and melanin synthesis (*tyrb*, *pmela*, *dct*) were significantly downregulated in both *ednrb1a*^−/−^ and double mutants, while genes inhibiting melanogenesis (*foxd3* and *asip1*) were significantly upregulated in double mutants [67,68,69,70]. For xanthophores, genes regulating differentiation (*pax3b*, *csf1ra*, *pax7a*, *pax7b*) and pigmentation (*scarb1*, *plin6*) showed significant downregulation in *ednrb1a*^−/−^ and double mutants, whereas genes related to carotenoid metabolism (*bco2b*, *bco1*, *bco2a*) exhibited marked upregulation (Figure 6C,D) [71,72,73,74,75]. At 150 dpfs *ednrb1a*^−/−^;*ednrb2*^−/−^ mutants exhibited substantially reduced pigment cells in scales and caudal fin compared to WT controls (Figure 6E).

### 3.10. Microinjection of mitfa mRNA but Not pnp4a mRNA Rescues Embryonic Phenotypes in ednrb1a^−/−^;ednrb2^−/−^ Mutants

**Figure 7:**
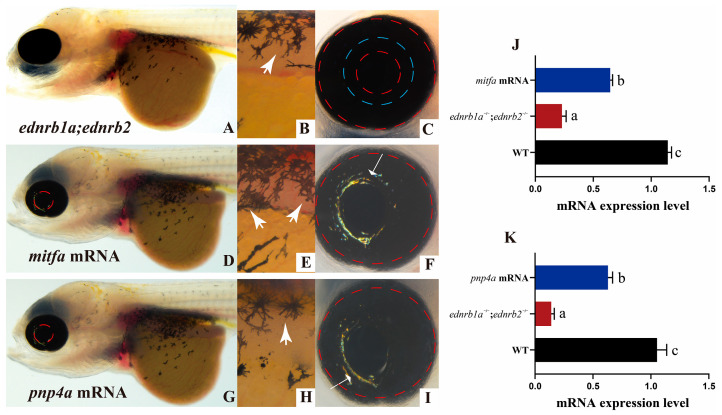
Functional rescue of *ednrb1a*^−/−^;*ednrb2*^−/−^ mutants at 7 dpfs via mRNA microinjection. (**A**,**D**,**G**) Whole-body views: (**A**) Uninjected mutant, (**D**) *mitfa* mRNA-injected, (**G**) *pnp4a* mRNA-injected. (**B**,**E**,**H**) Abdominal regions: Arrows indicate melanophore recovery. (**C**,**F**,**I**) Ocular regions: Arrowheads show iridophore restoration. (**J**,**K**) qPCR analysis: *mitfa* (**J**) and *pnp4a* (**K**) transcript levels. Data are presented as the mean ± SD; *p* < 0.05. Scale bars: 500 μm (**A**,**D**,**G**), 100 μm (**B**,**C**,**E**,**F**,**H**,**I**). Different letters above the error bar indicate statistical differences. *p* < 0.05 is considered to be sta-tistically significant. dpfs, days post fertilization.

The microinjection of *mitfa* or *pnp4a* mRNA into *ednrb1a*^−/−^;*ednrb2*^−/−^ mutants revealed partial rescue of iridophore phenotypes. At 7 dpfs, iridophore restoration was observed in ocular regions across rescue cohorts, with *mitfa* mRNA injection yielding superior iridophore recovery compared to *pnp4a* injection (Figure 7A–I). Notably, *mitfa*-rescued mutants exhibited significantly increased abdominal melanophores, while *pnp4a* rescue showed no such effect. However, this rescued phenotype was transient and completely diminished by 15 dpfs.

qPCR analysis confirmed transcriptional rescue efficiency: *mitfa* mRNA restored 50% of endogenous expression levels, and *pnp4a* mRNA restored 60% (Figure 7J,K). The partial rescue of iridophores by both *mitfa* and *pnp4a* mRNA injections provides preliminary functional evidence that these genes act as candidate downstream targets of Ednrb signaling, though further validation is required to confirm their regulatory hierarchy [76,77].

## 4. Discussion

In teleosts, all pigment cells, except those in RPE (non-neural crest-derived) cells, originate from multipotent NCCs. These NCCs differentiate, proliferate, and migrate to form mature pigment cells, a process that occurs during larval-to-adult transitions [78]. Melanophores and iridophores share a common precursor cell, while lineage tracing in zebrafish reveals that iridophore precursors migrate via the horizontal myoseptum to the subepidermal layer, forming primary iridophore stripes [79]. In tilapia *ednrb1a*^−/−^;*ednrb2*^−/−^ mutants, iridophores were absent from early development, indicating a failure of precursor differentiation. Concurrent reductions in melanophores, xanthophores, and erythrophores suggested that *ednrb1a* and *ednrb2* broadly regulate pigment cell specification, though they are indispensable only for iridophores. *ednrb2*^−/−^ mutants exhibited iridophore loss specifically in the dorsal iris, implicating *ednrb2* in iridophore precursor localization. This phenotype suggests a previously uncharacterized mechanism of pigment cell regulation in teleosts. Studies in zebrafish highlight genetic hierarchies governing pigment cell development: *mitfa* drives melanophore fate [80], while *tfec* [81] and *alx4a* [82] regulate iridophores differentiation.

In tilapia, *ednrb1a;ednrb2* mutants show downregulated *tfec*, *mpv17*, *alx4a*, and *pnp4a* in the skin, possibly modulating *tfec*-dependent iridophore differentiation, akin to Edn3-Ednrb-Mitf signaling in tetrapods [83].

In zebrafish, *ednrb1a* mutations reduce melanophores and iridophores only after 21 dpfs, prompting Parichy’s hypotheses of paralog compensation or precursor threshold effects [6]. However, in tilapia, *ednrb1a* failed to compensate for *ednrb2* loss, and no upregulation of *ednrb2* expression was observed in *ednrb1a* mutants, which refutes paralog compensation and implies synergistic roles in pigment cell maturation. Notably, *ednrb1a*^−/−^ tilapia exhibited early iridophore defects, in sharp contrast to zebrafish, where phenotypes emerged later. This discrepancy may stem from mutation type: tilapia *ednrb1a* frameshifts disrupt differentiation from onset, while zebrafish models retain partial function. Strikingly, *ednrb2*^−/−^ tilapia showed iridophore loss only in the dorsal iris, underscoring *ednrb1a*’s dominant role in pigment cell development. This functional hierarchy may explain why *ednrb2* was lost in certain mammalian and cypriniform lineages.

### 4.1. ednrb1a and ednrb2 Are Indispensable for Iridophore Development in Tilapia

In tilapia *ednrb1a*^−/−^*;ednrb2*^−/−^ mutants, iridophores were absent from the earliest developmental stages, indicating a failure in iridophore precursor differentiation. Concurrently, melanophores, xanthophores, and erythrophores were significantly reduced, demonstrating that *ednrb1a* and *ednrb2* participate in the development of these pigment cell precursors or their lineage specification but are not strictly required for these processes. However, they are indispensable for iridophore development. The mutation of *ednrb2* specifically abolished iridophores in the dorsal iris, suggesting that *ednrb2* is essential for iridophore precursor colonization in this region. To date, this provides initial functional evidence for *ednrb2* in iridophore formation in teleosts.

Studies on zebrafish color mutants have revealed genetic regulators of NCC-derived pigment cell development. Some genes, such as *sox10*, are essential for all pigment cell lineages (e.g., *sox10* mutants lack nearly all pigment cells), while others, like *mitfa*, specifically affect melanophores. The melanocyte regulatory network is the most well-characterized, with Mitfa identified as the “master regulator” of melanocyte fate in mammal [80]. In zebrafish, *mitfa* overexpression induces ectopic expression of melanophore precursor markers (e.g., *dct*) and xanthophore precursors (e.g., *gch2*). Double *mitfa*;*mitfb* mutants exhibit severe xanthophore loss in the trunk, though head xanthophores persist, suggesting compensatory pathways [74,75,84]. In medaka, *mitfa*;*mitfb* double mutants lose melanophores, xanthophores, and leucophores (iridophores remain unaffected), with in situ hybridization confirming that Mitfs directly activate *dct* and *gch2* transcription. Despite species-specific differences, Mitfs are critical for melanophore and xanthophore development. Given that xanthophores and erythrophores share a lineage, and tetrapod Ednrb regulates Mitf expression via Edn3 signaling, the observed *mitfa* downregulation in *ednrb1a*^−/−^ and *ednrb1a*^−/−^*;ednrb2*^−/−^ mutants (supported by qPCR data) likely disrupted melanophore, xanthophore, and erythrophore development, reducing their numbers [85,86,87].

In zebrafish, *tfec* acts as a key transcriptional regulator for iridophores, analogous to *mitfa* in melanophores; *tfec* mutation eliminates iridophores [81]. Similarly, zebrafish *mpv17* mutants exhibit translucency due to mitochondrial dysfunction impairing guanine deposition [88]. Nile tilapia *mpv17* mutants display comparable phenotypes, with guanine supplementation failing to rescue iridophore defects, suggesting abnormal precursor differentiation rather than apoptosis [89,90]. Medaka *pnp4a* mutants lack iridophores in larval eyes, but adults show partial recovery, indicating redundant nucleotide phosphorylases [85]. Zebrafish *alx4a* mutants lose body iridophores, highlighting their role in precursor differentiation [82]. As membrane receptors, *ednrb1a* and *ednrb2* contribute to iridophore precursor differentiation, though their interplay with *tfec*, *mpv17*, *alx4a*, and *pnp4a* remains unclear. The severe iridophore loss in tilapia *ednrb1a*^−/−^;*ednrb2*^−/−^ mutants, coupled with downregulated *tfec*, *mpv17*, *alx4a*, and *pnp4a* expression, suggests that Ednrb signaling enhances Tfec-mediated iridophore lineage specification and collaborates with other genes in iridophore development. Taken together, these results suggest that *ednrb1a* and *ednrb2* are indispensable for normal iridophore development in teleosts [91,92].

### 4.2. Synergistic Roles of ednrb1a and ednrb2 in Tilapia Pigment Cell Development

In zebrafish, *ednrb1a* mutation reduces melanophore and iridophore numbers, but this phenotype manifests only in later developmental stages, with juveniles appearing phenotypically normal and adults retaining half the wild-type pigment cell counts. Two hypotheses were proposed for this delayed effect: paralog compensation or a threshold requirement for precursor cell numbers [6]. However, our findings in tilapia contradict the paralog compensation hypothesis. In *ednrb2*^−/−^ mutants, *ednrb1a* expression failed to compensate for *ednrb2* loss, and *ednrb2* expression did not increase in *ednrb1a*^−/−^ mutants. Strikingly, *ednrb2* expression was even downregulated in the more severe *ednrb1a*^−/−^ mutant. These results suggest that *ednrb1a* and *ednrb2* act synergistically rather than redundantly during pigment cell development. While current data cannot confirm whether *ednrb1a* drives precursor proliferation, it is evident that both paralogs are essential for the migration and differentiation of all pigment cell lineages, as *ednrb1a*^−/−^*;ednrb2*^−/−^ mutants exhibit marked reductions or complete loss of melanophores, iridophores, xanthophores, and erythrophores [93].

Notably, the *ednrb1a*^−/−^ tilapia mutant displayed early-onset iridophore depletion in the iris, contrasting with zebrafish *ednrb1a* mutants, where pigment cell defects emerge later. This divergence may reflect species-specific mutation effects: tilapia *ednrb1a* mutations disrupted iridophore differentiation from the onset, whereas zebrafish phenotypes arise post-threshold. Intriguingly, *ednrb2*^−/−^ mutants lost iridophores only in the dorsal iris, with minimal impact on body pigmentation, whereas *ednrb1a* mutants exhibited more severe pan-pigment cell defects. This indicates that *ednrb1a* exerts a stronger regulatory influence on pigment cell development than *ednrb2*, likely due to its dominant role in endothelin signaling [94]. The weaker functional contribution of *ednrb2* may explain its secondary loss in certain mammalian and cypriniform lineages.

To date, no prior studies in fish have concurrently investigated both endothelin receptor paralogs. Dual-color fluorescence in situ hybridization demonstrated the co-expression of *ednrb1a* and *ednrb2* in NCCs and ocular tissues in tilapia. This expression pattern exhibits phenotypic concordance with knockout models: single mutants (*ednrb1a*^−/−^ or *ednrb2*^−/−)^ displayed incomplete iridophore loss in ocular regions, whereas double mutants (*ednrb1a*^−/−^*;ednrb2*^−/−^) exhibited a complete absence of iridophores. Crucially, while single mutants showed no extensive depletion of pigment cells, double mutants manifested near-complete transparency. These results demonstrate synergistic roles of *ednrb1a* and *ednrb2* in regulating the differentiation and migration of pigment cells and their NCC progenitors, providing mechanistic insights into Ednrb2 function in chromatophore development [95].

Real-time PCR analysis demonstrated antagonistic relationships within pigment regulatory networks: in melanophore-associated genes, *foxd3* antagonizes *mitfa*, while *mc1r* opposes *asip1*; concurrently, for carotenoid metabolism, the receptor *scarb1* exhibits functional antagonism against the cleavage enzymes *bco1*, *bco2b*, and *bco2a*. These regulatory dynamics align with established mechanisms in zebrafish. Complementarily, pigment cell differentiation/migration genes were downregulated in *ednrb1a*^−/−^ or *ednrb2*^−/−^ mutants. Crucially, *ednrb1a*^−/−^*;ednrb2*^−/−^ mutants showed significantly stronger suppression than the additive effect of individual mutants, indicating non-additive genetic interaction. This synergistic suppression confirms cooperative roles of *ednrb1a* and *ednrb2* in regulating the differentiation and migration of pigment cells and their neural crest-derived progenitors—consistent with the enhanced phenotypic severity (near-complete transparency) in double knockouts.

### 4.3. ednrb1a and ednrb2 Regulate Erythrophore Development

In zebrafish, *ednrb1a* is enriched in xanthophores, and its knockout results in the partial depletion of this pigment cell population. Notably, zebrafish lack erythrophores, a chromatophore type present in tilapia. *ednrb1a*^−/−^*;ednrb2*^−/−^ mutants exhibit severe loss of both erythrophores and xanthophores, accompanied by significant downregulation of xanthophore differentiation genes (*csf1ra*, *pax3b*, *pax7a*, *pax7b*). These findings identify *ednrb* as a putative key regulator for erythrophore differentiation and migration and demonstrate shared lineage commitment between erythrophores and xanthophores.

### 4.4. Ednrb Mutants Do Not Affect the Guanine Synthesis Pathway in Tilapia

In zebrafish studies of *ednrb1a* mutants [6], the focus has been on iridophore differentiation, while the specific mechanism underlying iridophore defects, whether due to differentiation failure or impaired coloration, remains unresolved.

In this study, we established ednrb mutant lines in tilapia. The analysis of guanine content in the skin of *ednrb* mutant larvae at 30dpfs revealed a distinct phenotype compared to *tfec* and *mpv17* mutants, which also exhibit iridophore loss. While *ednrb* mutants displayed a similar loss of iridophores, their cutaneous guanine levels showed no significant reduction. This demonstrated that the guanine demand of iridophores constitutes a minor fraction of total skin guanine content, and *ednrb* deficiency-induced apoptosis of iridophore precursor cells does not impair guanine synthesis pathways. The chromatophore-specific pigment in iridophores consists of guanine crystals, whose abundance correlates directly with the number and packing architecture of reflecting platelets. Therefore, quantifying cutaneous guanine content provides a necessary metric for assessing iridophore alterations. These findings imply that iridophores themselves require minimal guanine crystal deposition. Notably, as guanine is a fundamental component of nucleic acids, its severe depletion could lead to sterility. Leveraging this insight, we successfully generated a novel, fertile transparent Nile tilapia mutant, contributing to fish germplasm innovation and enriching aquatic genetic resources.

### 4.5. mitfa mRNA Injection Successfully Rescues the Pigment Cell Deficiency Phenotype in ednrb1a^−/−^;ednrb2^−/−^ Mutants

In tetrapods, the Ednrb signaling pathway is well-characterized as the canonical Edn3-Ednrb-Mitf cascade [83], where the transcription factor Mitf functions as the core downstream target of Ednrb, governing melanocyte differentiation, migration, and pigment synthesis to orchestrate vertebrate melanogenesis. Notably, no studies in teleosts have previously reported functional rescue of *ednrb* deficiencies using *mitf* genes. Here, we demonstrate the first successful partial functional rescue of melanophore and iridophore loss in *ednrb1a*^−/−^;*ednrb2*^−/−^ mutants via *mitfa* mRNA microinjection. This breakthrough establishes (1) *mitfa* as an essential functional component of the Ednrb pathway in fish, and (2) evolutionary conservation of endothelin signaling in vertebrate pigment cell regulation. However, whether *mitfa* acts as a direct transcriptional target of Ednrb (analogous to mammalian Mitf–Ednrb interactions) requires further validation, while rescue attempts with *pnp4a* mRNA proved significantly less effective than *mitfa*.

*pnp4a* is established as a specific marker gene for iridophores. Cross-species studies in zebrafish, koi carp, and tilapia confirm its tissue-specific enrichment in iridophore-rich structures (e.g., dermal reflective layers and iris), with expression strongly correlating with guanine crystal deposition [96,97]. Functional analyses reveal that *pnp4a* knockdown disrupts iridophore ultrastructure, manifesting as disorganized crystal arrays, demonstrating its essential role in regulating guanine metabolism to mediate light-reflective function [98].

Notably, the microinjection of *pnp4a* mRNA into *ednrb1a*^−/−^*;ednrb2*^−/−^ mutants induced partial restoration of ocular iridophores at 7 dpfs, though rescue efficacy was significantly weaker than *mitfa*-mediated recovery. This limited rescue capacity underscores metabolic constraints imposed by guanine pathway complexity.

Importantly, *pnp4a* overexpression in zebrafish yielded only localized enhancement of dermal reflectivity without increasing iridophore numbers [55,72], suggesting its primary action involves the functional modification of existing iridophores rather than de novo cell generation.

## 5. Conclusions

This study demonstrates that *ednrb1a* and *ednrb2* play essential and synergistic roles in pigment cell development in Nile tilapia. Specifically, both receptors are indispensable for the differentiation of iridophore precursor cells, with *ednrb2* being critically required for iridophore colonization in the dorsal iris. Mutations in these genes lead to the severe depletion of iridophores, melanophores, xanthophores, and erythrophores, resulting in a translucent body phenotype. Crucially, these mutations do not impair guanine synthesis pathways, indicating that the iridophore defect stems from failed precursor differentiation rather than disrupted nucleotide metabolism. Furthermore, the successful partial rescue of pigment cell deficiencies in double mutants via *mitfa* mRNA microinjection establishes *mitfa* as a key functional component downstream of the Ednrb signaling pathway in teleosts. These findings provide significant insights into the evolutionary conservation and functional divergence of endothelin receptors in vertebrate pigment cell regulation and offer a novel strategy for generating valuable transparent germplasm in aquaculture species.

## Data Availability

The original contributions presented in this study are included in the article and Supplementary Materials. Further inquiries can be directed to the corresponding authors.

## Supplementary Materials

The following supporting information can be downloaded at: https://www.mdpi.com/article/10.3390/cells14151213/s1. Figure S1. Alignment of amino acid sequences of Ednrb in vertebrates. Figure S2. Phylogenetic tree of Ednrb in vertebrates. Figure S3. Expression of ednrb in tilapia embryos by WISH. Figure S4. Target selection and establishment of tilapia ednrb1a,ednrb2 and ednrb1a;ednrb2 homozygous mutant line. Figure S5. Histological analysis of iris to show the abnormal pigmentation in ednrb1a and ednrb2 mutants. Figure S6. Comparative abdominal morphology at 30 dpfs: WTs versus ednrb1a−/−;ednrb2−/− mutants. Table S1. The accession numbers of sequences used in multiple alignment and phylogenetic. Table S2. Primer sequences used in the present study.
