# Supplementary material for: Synergistic Regulation of Pigment Cell Precursors’ Differentiation and Migration by ednrb1a and ednrb2 in Nile Tilapia
Source: Cells. 2025 Aug 6;14(15):1213. doi: 10.3390/cells14151213 (PMC12346172; doi:10.3390/cells14151213)
Supplement: Supplementary file 1 [file cells-14-01213-s001.zip › cells-3780226-supplementary.pdf]

Supplementary materials

Table S1. The accession numbers of sequences used in multiple alignment and phylogenetic.

Gene	English name	Latin name	Accession number
EDNRB	Human	*Homo sapiens*	NP_000106.1
Ednrb	House mouse	*Mus musculus*	NP_001129533.1
Ednrb	Tammar wallaby	*Notamacropus eugenii*	XP_072478271.1
Ednrb1	Australian echidna	*Tachyglossus aculeatus*	XP_038614731.1
Ednrb1	Platypus	*Ornithorhynchus anatinus*	XP_028929581.1
Ednrb1	Chicken	*Gallus gallus*	NP_001001127.1
Ednrb1	Common lizard	*Zootoca vivipara*	XP_034972028.2
ednrb1	Mississippi paddlefish	*Polyodon spathula*	XP_041115639.1
ednrb1	Spotted gar	*Lepisosteus oculatus*	XP_006639127.1
ednrb1a	Yellow catfish	*Tachysurus fulvidraco*	XP_027012260.1
ednrb1a	Electric eel	*Electrophorus electricus*	XP_026867628.1
ednrb1a	Red-bellied piranha	*Pygocentrus nattereri*	XP_017565589.1
ednrb1a	Tambaqui	*Colossoma macropomum*	XP_036413605.1
ednrb1a	Nile tilapia	*Oreochromis niloticus*	XP_003453339.1
ednrb1a	Japanese medaka	*Oryzias latipes*	XP_004079054.1
ednrb1a	Torafugu	*Takifugu rubripes*	XP_029686414.1
ednrb1a	Grass carp	*Ctenopharyngodon idella*	XP_051752887.1
ednrb1a	Goldfish	*Carassius auratus*	XP_026117971.1
ednrb1a	Zebrafish	*Danio rerio*	NP_571272.1
ednrb1a	Three-spined stickleback	*Gasterosteus aculeatus*	XP_040028054.1
ednrb1aa	Razorback sucker	*Xyrauchen texanus*	XP_051981716.1
ednrb1ab	Razorback sucker	*Xyrauchen texanus*	XP_051996653.1
ednrb1aa	Chinese sucker	*Myxocyprinus asiaticus*	XP_051565863.1
ednrb1ab	Chinese sucker	*Myxocyprinus asiaticus*	XP_051501753.1
ednrb1aa	Rainbow trout	*Oncorhynchus mykiss*	XP_021469528.1
ednrb1ab	Rainbow trout	*Oncorhynchus mykiss*	XP_036822586.1
ednrb1b	Nile tilapia	*Oreochromis niloticus*	XP_003455495.2
ednrb1b	Japanese medaka	*Oryzias latipes*	XP_004081995.1
ednrb1b	Torafugu	*Takifugu rubripes*	XP_029697833.1
ednrb1b	Grass carp	*Ctenopharyngodon idella*	XP_051762018.1
ednrb1b	Goldfish	*Carassius auratus*	XP_026073169.1
ednrb1b	Zebrafish	*Danio rerio*	XP_688565.1
ednrb1b	Large-scale loach	*Paramisgurnus dabryanus*	XP_065107492.1
ednrb1b	Razorback sucker	*Xyrauchen texanus*	XP_051997809.1
ednrb1b	Chinese sucker	*Myxocyprinus asiaticus*	XP_051565506.1
ednrb1b	Three-spined stickleback	*Gasterosteus aculeatus*	XP_040057098.1
ednrb1b	Red-bellied piranha	*Pygocentrus nattereri*	XP_017574877.2
ednrb1a	Tambaqui	*Colossoma macropomum*	XP_036417874.1
ednrb1b	Rainbow trout	*Oncorhynchus mykiss*	XP_036821119.1
Ednrb2	Platypus	*Ornithorhynchus anatinus*	XP_007669762.1
Ednrb2	Australian echidna	*Tachyglossus aculeatus*	XP_038603782.1
Ednrb2	Chicken	*Gallus gallus*	NP_989451.1
Ednrb2	Common lizard	*Zootoca vivipara*	XP_034969702.1
ednrb2	Mississippi paddlefish	*Polyodon spathula*	XP_041111706.1
ednrb2	Yellow catfish	*Tachysurus fulvidraco*	XP_026991060.1
ednrb2	Electric eel	*Electrophorus electricus*	XP_026860686.2
ednrb2	Red-bellied piranha	*Pygocentrus nattereri*	XP_017579023.1
ednrb2	Tambaqui	*Colossoma macropomum*	XP_036425305.1
ednrb2	Spotted gar	*Lepisosteus oculatus*	XP_006633024.2
ednrb2	Nile tilapia	*Oreochromis niloticus*	XP_013119897.1

ednrb2	Japanese medaka	*Oryzias latipes*	NP_001098314.1
ednrb2	Torafugu	*Takifugu rubripes*	XP_003970164.2
ednrb2	Three-spined stickleback	*Gasterosteus aculeatus*	XP_040030735.1
ednrb2	Rainbow trout	*Oncorhynchus mykiss*	XP_036799504.1
nmbr	Nile tilapia	*Oreochromis niloticus*	XP_005452510.1

Table S2. Primer sequences used in the present study.

**Primer**	**Sequence （5'-3'）**
ednrb1a-Real-time PCR-F	TTAAAACAGCGGAGGGAGGT
ednrb1a-Real-time PCR-R	TTCATCATAGATGGTGAGCTTCAG
ednrb1b-Real-time PCR-F	AGGCTCATGGCAGAAAATTGG
ednrb1b-Real-time PCR-R	TGTCAACACTCAAGGCACAT
ednrb2-Real-time PCR-F	GAAGCAGAGGAGGGAAGTAGC
ednrb2-Real-time PCR-R	GGGTCATCCTCCTCATAGACTG
gapdh-Real-time PCR-F	TCCCTCAAGGTTGTCAGCAAT
gapdh-Real-time PCR-R	GTGCTCATCAGGCCCTCAAT
mitfa-q-F / *mitfa*-q-R	CCCCAATCAGAAGCAGTGGT / CTTGTAGAGGCCATGCTGGT
kita-q-F / *kita*-q-R	CCCACCTGGCTCGCAAC / GCATCGATTTGCAGTGTCGT
kitlga-q-F / *kitlga*-q-R	TTCCTCCCTGTCCCACCTC / GGAGTCTTTGTTGCTGACTTGT
aspi1-q-F / *asip1*-q-R	GCCTGGCAAACACAGAAAGAT / TCATCTGGGATCATGTGGGC
mc1r-q-F / *mc1r*-q-R	CCGTTCAGCTCTCTCCCAAG / TGCTGCGTCCTGAATGACTT
foxd3-q-F / *foxd3*-q-R	AGCCCAAGAGTAGCCTGGTA / TGATGAACTCGCAGATCCCG
csf1ra-q-F / *csf1ra*-q-R	CAGGCGCATTCGGAAAAGTT / GCACTGGAGTGTAACAGTGGA
pax3b-q-F / *pax3b*-q-R	AGAAGTAGAGTCGGAGCCTGAT / CCTGTTGCTGAACCACACCT
pax7a-q-F / *pax7a*-q-R	CAAGAGAGAAAACCCGGGCA/ CTGTCCGGTTACCTGAAGGA
pax7b-q-F / *pax7b*-q-R	AGCTGGGAGATCCGGGATAA/ CGGCTGATCGAACTCACAGA
mpv17-q-F / *mpv17*-q-R	GCCTTTGGAGAGGCTACCAG / CGACACCCACTAAAGACCCC
alx4a-q-F / *alx4a*-q-R	GTTTGGTCAACGACAGTGCT / AACGCAGGTCTCAGTGTTCAT
tfec-q-F / *tfec*-q-R	GCCAATCATGACAGTGAGGTG / TCGTGTTCTTGTTCACCCCCAT
tyrb-q-F / *tyrb*-q-R	GGCACAAGAGATCTGGCAGT / CGCTTCCATTTCCGTCTTGC
dct-q-F / *dct*-q-R	ACATGATTTGGCTGCAGGAGT / GCTACGGTTCCTTGCTGTGT
scarb1-q-F / *scarb1*-q-R	TGTCTCTCAAGGCAGAACAAGA / GGGTTTAGACTGGCAACTGGA
plin6-q-F / *plin6*-q-R	CCCAGGAGCGCTCTTTATCTA / TTCTCTTTCGCCAAATCCCA
pnp4a-q-F / *pnp4a*-q-R	TCTGCAAGACGACCTTTCCC / CAGTGAGCCAGCAGCATTTG
gart-q-F / *gart*-q-R	CACCCTATGTGGGTGTGCTG / CACCTGACATTCGGGGTCTC
atic-q-F / *atict*-q-R	AGAACTGGATCAGCTCACTGC/ TATTGGACGCCGCTCCTTTT
aprt-q-F / *aprtt*-q-R	CCAGAGGTTGAGGCTTCGTT/ GCCTTTATCGCCCTCTTTTTGA

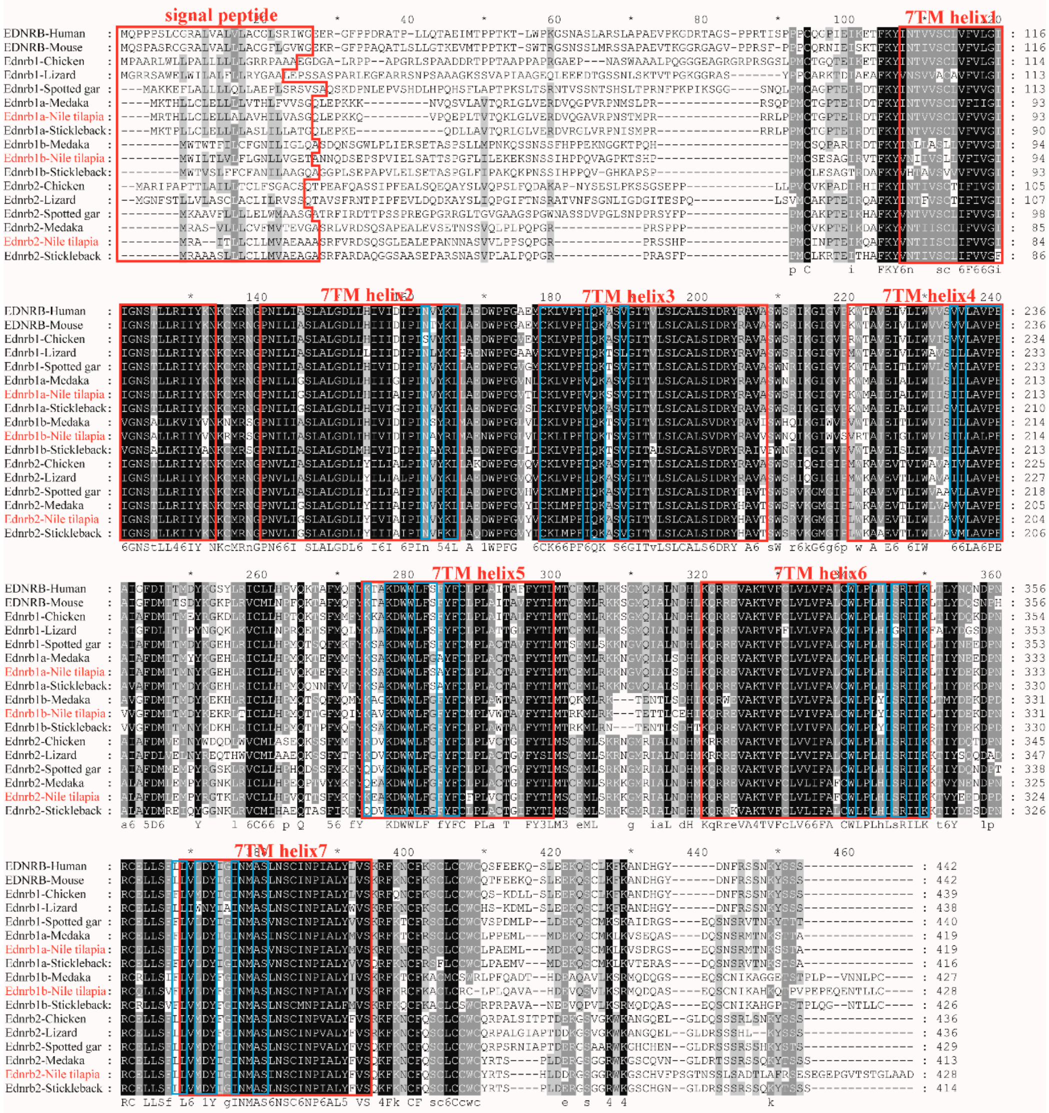

Figure S1. Alignment of amino acid sequences of Ednrb in vertebrates. The diagram of the alignment was drawn by GeneDoc software. The black area represents the conserved amino acids. The red boxes represent signal peptides and the seven transmembrane helical domains of G protein-coupled receptors. The blue boxes represent ligand binding sites. The results showed that the Ednrb amino acid sequences of these species were highly conserved. 7 TM, seven transmembrane domains.

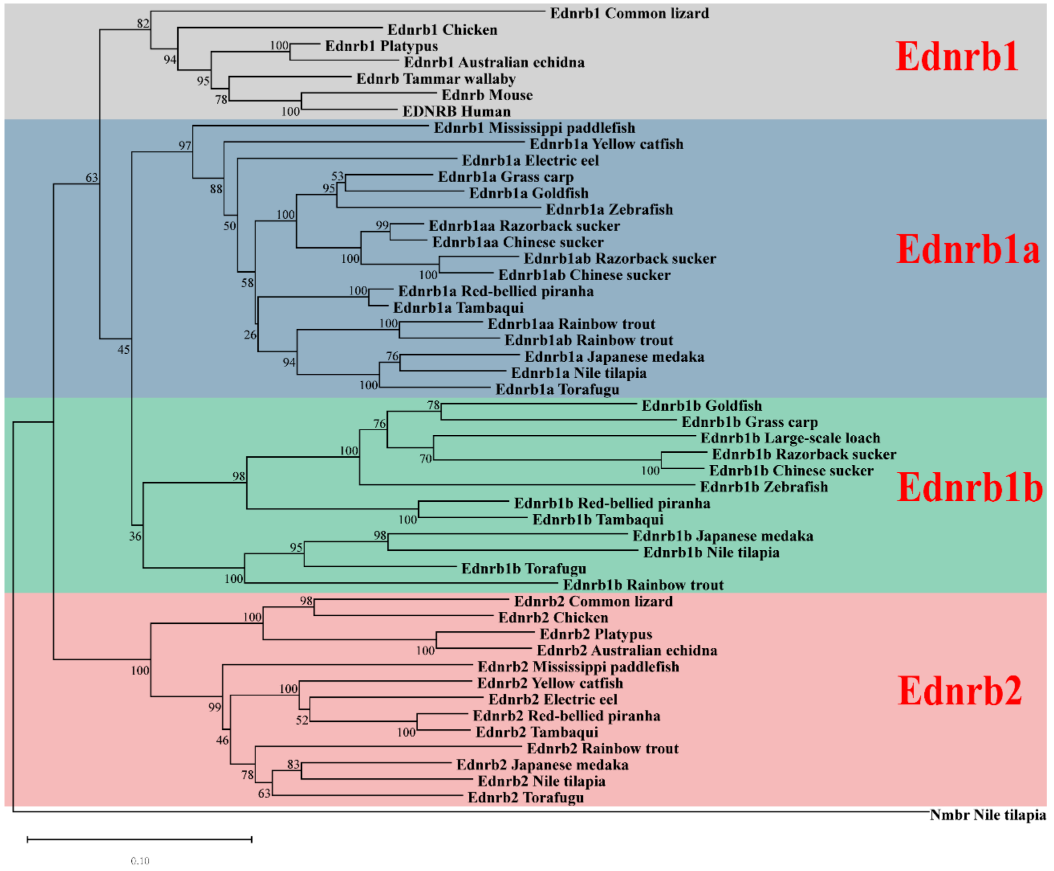

Figure S2. Phylogenetic tree of Ednrb in vertebrates. Phylogenetic analysis of Ednrb in vertebrates. The phylogenetic tree was constructed in MEGA-X software, where the Ednrab of tilapia was used as an outgroup, and multiple amino acid sequence alignments were performed using ClustalW. The phylogenetic tree was constructed using Neighbor-Joining method, and the numbers on the branching points of the tree were bootstrap value.

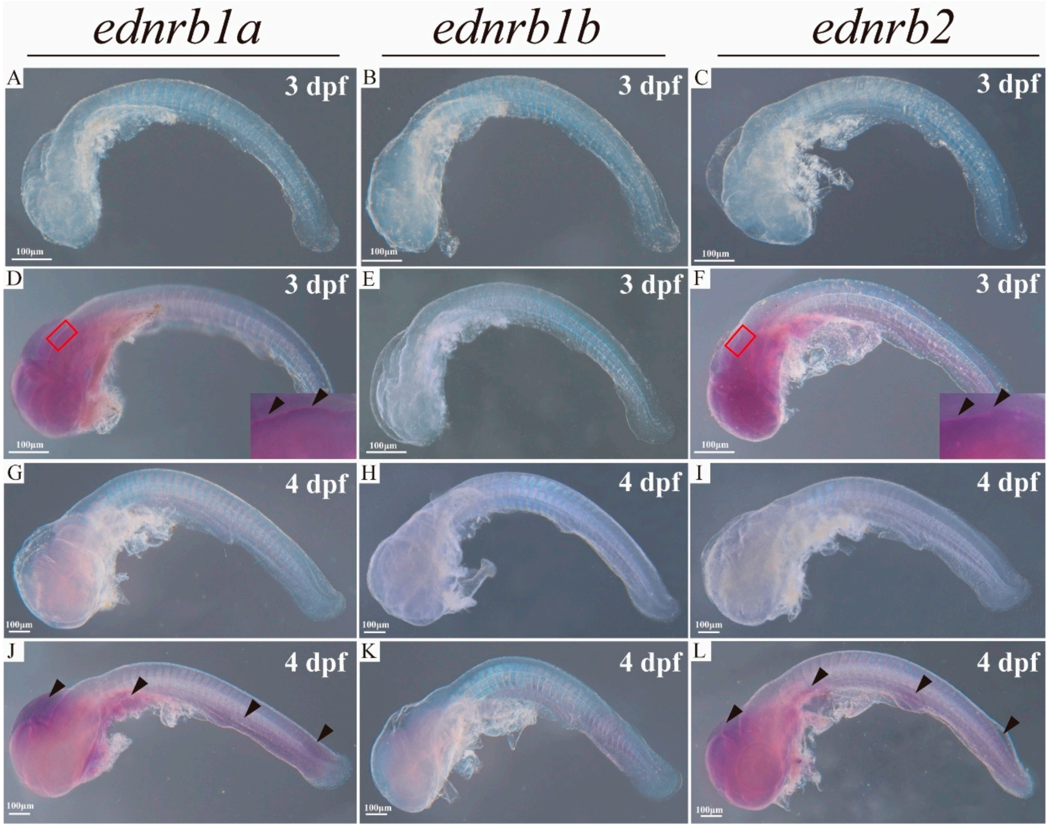

Figure S3. Expression of *ednrb* in tilapia embryos by WISH. A-C, G-I) Negative controls with *ednrb1a*, *ednrb1b* and *ednrb2* sense probes. D-F) Expression of *ednrb1a*, *ednrb1b* and *ednrb2* in embryos at 3 dpf. The black arrows indicate the premigrating NCCs. J-L) Expression localization of *ednrb1a*, *ednrb1b* and *ednrb2* in embryos at 4 dpf. The black arrows indicate the migrating and differentiating NCCs.

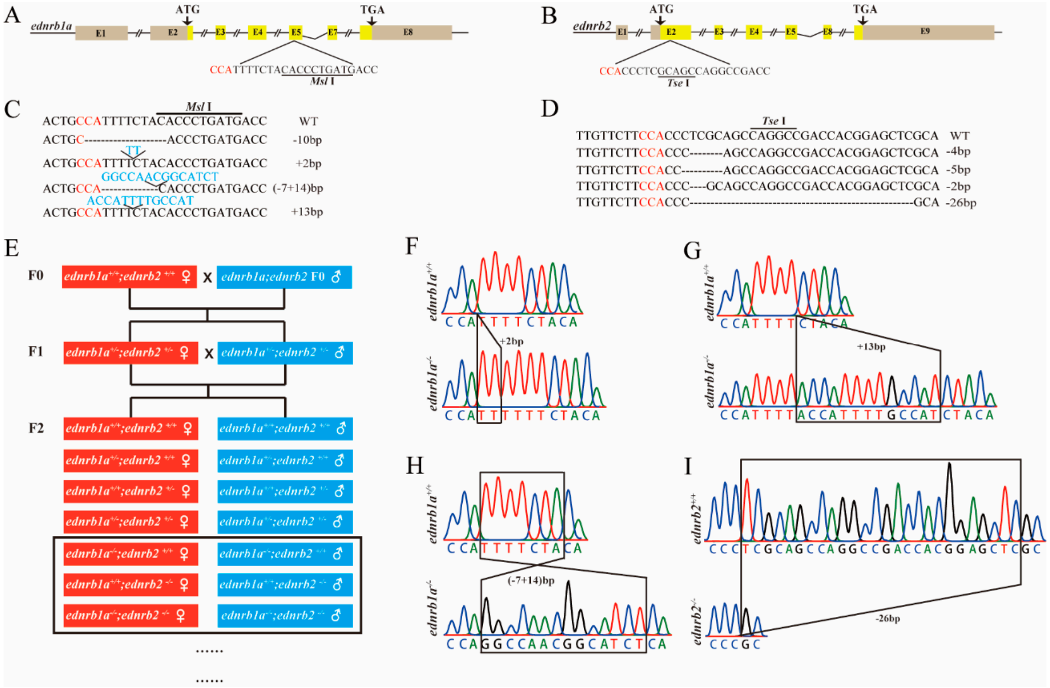

Figure S4. Target selection and establishment of tilapia ednrb1a, ednrb2 and ednrb1a; ednrb2 homozygous mutant line. A, B) Gene structures of *ednrb1a* and *ednrb2*, showing PAM sequences and the target sites with *Msl* I and *Tse* I restriction enzyme sites, respectively. C, D) Nucleotide sequences of the F0 mutants were obtained by Sanger sequencing. Blue sequences represent nucleotide insertions and short dashes represent nucleotide deletions. E) The establishment processes of *ednrb1a*$^{-/-}$, *ednrb2*$^{-/-}$ and *ednrb1a*$^{-/-}$;*ednrb2*$^{-/-}$ mutants. F-H) Sequencing results of the wild type and *ednrb1a*$^{-/-}$ mutants with three different mutation types. I) Sequencing results of the wild type and *ednrb2*$^{-/-}$ mutants.

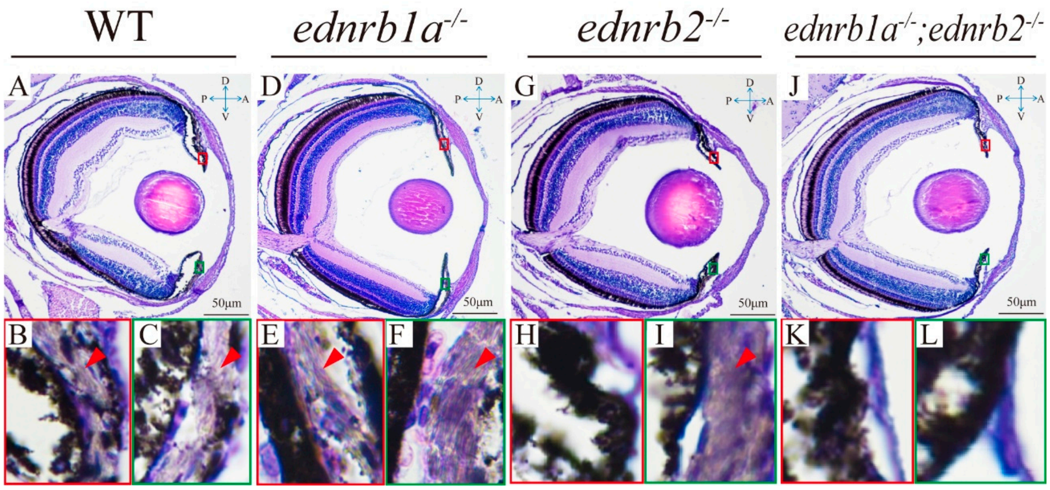

Figure S5. Histological analysis of iris to show the abnormal pigmentation in *ednrb1a* and *ednrb2* mutants. A-L) H.E. staining of eye in WT, *ednrb1a*$^{-/-}$, *ednrb2*$^{-/-}$ and *ednrb1a*$^{-/-}$;*ednrb2*$^{-/-}$ mutants. The red box is the magnified view of the dorsal iris, and the green box is the magnified view of the ventral iris. Red arrows indicate the area of abnormal pigmentation in iris.

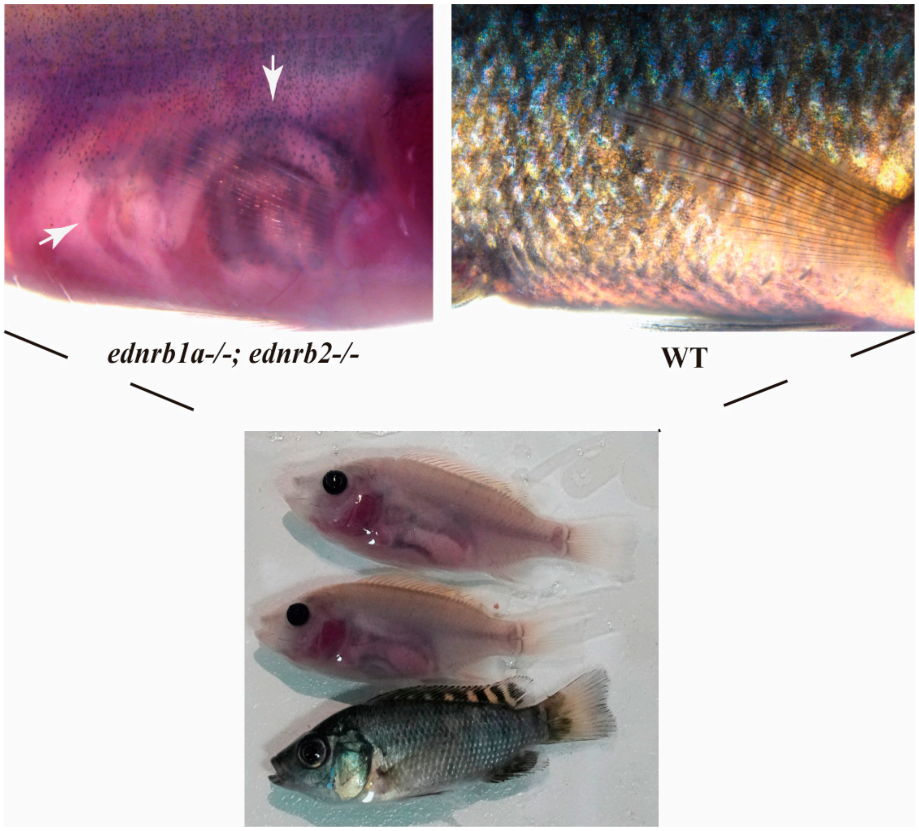

Figure S6. Comparative abdominal morphology at 30 dpf: WT versus ednrb1a$^{-/-}$;ednrb2$^{-/-}$ mutants. ednrb1a$^{-/-}$;ednrb2$^{-/-}$ mutants were transparent with internal organs visible due to complete iridophore loss on the body and iris
